# High prevalence of ascending aortic dilatation in a consecutive coronary CT angiography patient population

**DOI:** 10.1007/s00330-019-06433-z

**Published:** 2019-09-16

**Authors:** S. Petteri Kauhanen, Petri Saari, Pekka Jaakkola, Miika Korhonen, Johannes Parkkonen, Juska Vienonen, Ritva Vanninen, Timo Liimatainen, Marja Hedman

**Affiliations:** 1 Doctoral Programme of Clinical Research, University of Eastern Finland, Kuopio, Finland; 2 Department of Clinical Radiology, Clinical Imaging Center, Kuopio University Hospital, PO Box 100, Puijonlaaksontie 2, 70029 Kuopio, KYS Finland; 3 Department of Heart and Thoracic Surgery, Heart Center, Kuopio University Hospital, Kuopio, Finland; 4 School of Medicine, Clinical Radiology, University of Eastern Finland, Kuopio, Finland; 5 Research Unit of Medical Imaging, Physics and Technology, University of Oulu, Oulu, Finland; 6 Department of Diagnostic Radiology, Oulu University Hospital, Oulu, Finland

**Keywords:** Ascending aorta, Dilatation, Prevalence, Body surface area

## Abstract

**Objectives:**

To clarify the prevalence and risk factors of ascending aortic (AA) dilatation according to ESC 2014 guidelines.

**Methods:**

This study included 1000 consecutive patients scheduled for diagnostic coronary artery computed tomographic angiography. AA diameter was retrospectively measured in 3 planes: sinus valsalva, sinotubular junction, and tubular part. The threshold for AA dilatation was set to > 40 mm which has been suggested as an upper normal limit for AA diameter in ESC 2014 guidelines on aortic diseases. Aortic size index (ASI) using the ratio between aortic diameter and body surface area (BSA) was applied as a comparative measurement. The threshold for AA dilatation was set to the upper limit of normal distribution exceeding two standard deviations (95%). Risk factors for AA dilatation were collected from medical records.

**Results:**

The patients’ mean age was 52.9 ± 9.8 years (66.5% women). The prevalence of AA dilatation was 23.0% in the overall study population (52.5% males) and 15.1% in the subgroup of patients with no coronary artery disease or bicuspid (BAV)/mechanical aortic valve (*n* = 365). According to the normal-distributed ASI values, the threshold for sinus valsalva was defined as 23.2 mm/m^2^ and for tubular part 22.2 mm/m^2^ in the subgroup. Higher BSA was associated with larger AA dimensions (*r* = 0.407, *p* < 0.001). Male gender (*p* < 0.001), BAV (*p* < 0.001), hypertension (*p* = 0.009) in males, and smoking (*p* < 0.001) appeared as risk factors for AA dilatation.

**Conclusions:**

The prevalence of AA dilatation is high with current ESC guidelines for normal AA dimension, especially in males. Body size is strongly associated with AA dimensions; it would be more reliable to use BSA-adjusted AA diameters for the definition of AA dilatation.

**Key Points:**

• *The prevalence of AA dilatation is high in patients who are candidates for coronary CT angiography.*

• *Body size is strongly associated with AA dimensions.*

## Introduction

The European Society of Cardiology (ESC) guidelines suggest normal ascending aortic (AA) dimensions to be 40 mm or less in healthy adults. According to these criteria, patients with AA over 40 mm accompanied with risk factors should be monitored regularly either by computed tomography (CT) or magnetic resonance imaging (MRI) [1]. The recommended limit for surgical intervention in AA is 55 mm in cases with a normal aortic valve anatomy without inherited aortic disease [1, 2]. It has been demonstrated that the risk of aortic dissection or rupture increases considerably above this value [3].

The diameter of thoracic aorta and thoracic aortic dilatation (TAD) have been associated with increased age, male gender, and increased body surface area (BSA) [1]. Furthermore, it has been related to hypertension and smoking [4–6]. The hemodynamic conditions in the aorta play a significant role in determining TAD [7]. In particular, the bicuspid aortic valve (BAV) often causes valvular dysfunction (stenosis and regurgitation) and abnormal flow in the ascending aorta. BAV is associated strongly with TAD [8]. The prevalence of BAV in Western and Caucasian populations has varied from 0.5 to 10.9% depending on the study and study population [9–11]. Furthermore, the prevalence of TAD has been reported to range from 30% up to even 70% in patients with BAV [12–14].

The aim of this study was to determine the prevalence of AA dilatation according to ESC 2014 guidelines and to clarify AA dilatation risk factors in a consecutive single-center population scheduled for coronary CT angiography (CCTA). Our secondary aim was to compare ESC guidelines’ stratifications with a classification adjusted for the patient’s body size.

## Materials and methods

### Patient population

This retrospective study examined 1065 consecutive patients with low to moderate pretest probability for coronary artery disease (CAD) and without pre-existing aortic diseases who had been imaged with CCTA between January 2012 and March 2018. Sixty-four patients were excluded due to motion artifacts or inadequate visibility of AA in CCTA and one patient due to age under 16 years. Patients’ baseline characteristics are presented in Table 1.

**Table 1:** Baseline characteristics of the overall study population

	All patients (*n* = 1000)	Males (*n* = 335)	Females (*n* = 665)
Age (years)	52.9 ± 9.8	48.5 ± 10.8	55.1 ± 8.5
Height (cm)	168.7 ± 9.6	178.3 ± 6.3	163.6 ± 6.6
Weight (kg)	80.1 ± 17.7	90.7 ± 16.1	74.3 ± 15.8
BSA (m^2^)	1.9 ± 0.2	2.1 ± 0.2	1.8 ± 0.2
Diabetes	80 (8.0)	30 (9.0)	50 (7.5)
Hypertension	455 (45.5)	143 (42.7)	312 (46.9)
Hypercholesterolemia	500 (50.0)	160 (47.8)	340 (51.1)
Positive family history for CAD	572 (57.2)	168 (50.1)	404 (60.8)
Smoking	254 (25.4)	123 (36.7)	131 (19.7)
Normal CCTA	625 (62.5)	180 (53.7)	445 (66.9)
Over 50% stenosis in CCTA	149 (14.9)	55 (16.4)	94 (14.1)
Coronary calcification in CCTA	226 (22.6)	100 (29.9)	126 (18.9)
Bicuspid aortic valve	31 (3.1)	22 (6.6)	9 (1.4)
Mechanical aortic valve	1 (0.1)	0	1 (0.2)

*Abbreviations*: *BSA*, body surface area; *CAD*, coronary artery disease; *CCTA*, coronary computed tomography angiography

From this total study population (*n* = 1000), we selected for further analyses a subgroup with (1) no CAD (over 50% stenosis or coronary calcification) in CCTA, (2) no history of hypertension, or (3) no BAV or mechanical aortic valve. We named the subgroup “subgroup of patients with no risk factors.”

### CCTA imaging procedure

CCTA imaging was performed during mid-diastole according to routine clinical practice using four different CT scanners capable of ECG-gated fast coronary imaging (Somatom Definition AS 64; Somatom Definition AS+ 128; Definition Edge; Definition Flash, Siemens Medical Solutions). The slice thickness of 0.6 mm was used in all scanners. Collimation was 64 × 0.6 mm with the Somatom Definition AS 64, and 128 × 0.6 mm for the other scanners [15]. The patients were scanned in the supine position with their hands above their head to avoid artifacts. With 64- and 128-slice scanners, bolus tracking was used to optimize the timing of the coronary scanning. When using the dual-energy scanner (Definition Flash), a 10-ml contrast agent test bolus was injected prior to actual imaging to evaluate the optimal timing for coronary scanning. Depending on the scanner, the contrast agent volume varied between 60 and 80 ml (Omnipaque 350 mg/ml, GE Healthcare). The infusion rate was 5 ml/s followed by 30 ml of a saline chaser. The tube voltage was adjusted according to the patient’s size, varying between 80 and 120 kV. Tube current modulation was applied for every patient. The image area extended from the tracheal bifurcation to the inferior cardiac apex. The in-plane resolution was 512 × 512 pixels, with z-axis coverage including the area from the bifurcation to the diaphragm. Prospective ECG gating was applied during helical scanning. The heart rate was optimized to be below 65 beats/min by administering 5–20 mg metoprolol succinate intravenously (Seloken, AstraZeneca AB) [15].

### Data assessment

**Fig. 1:**
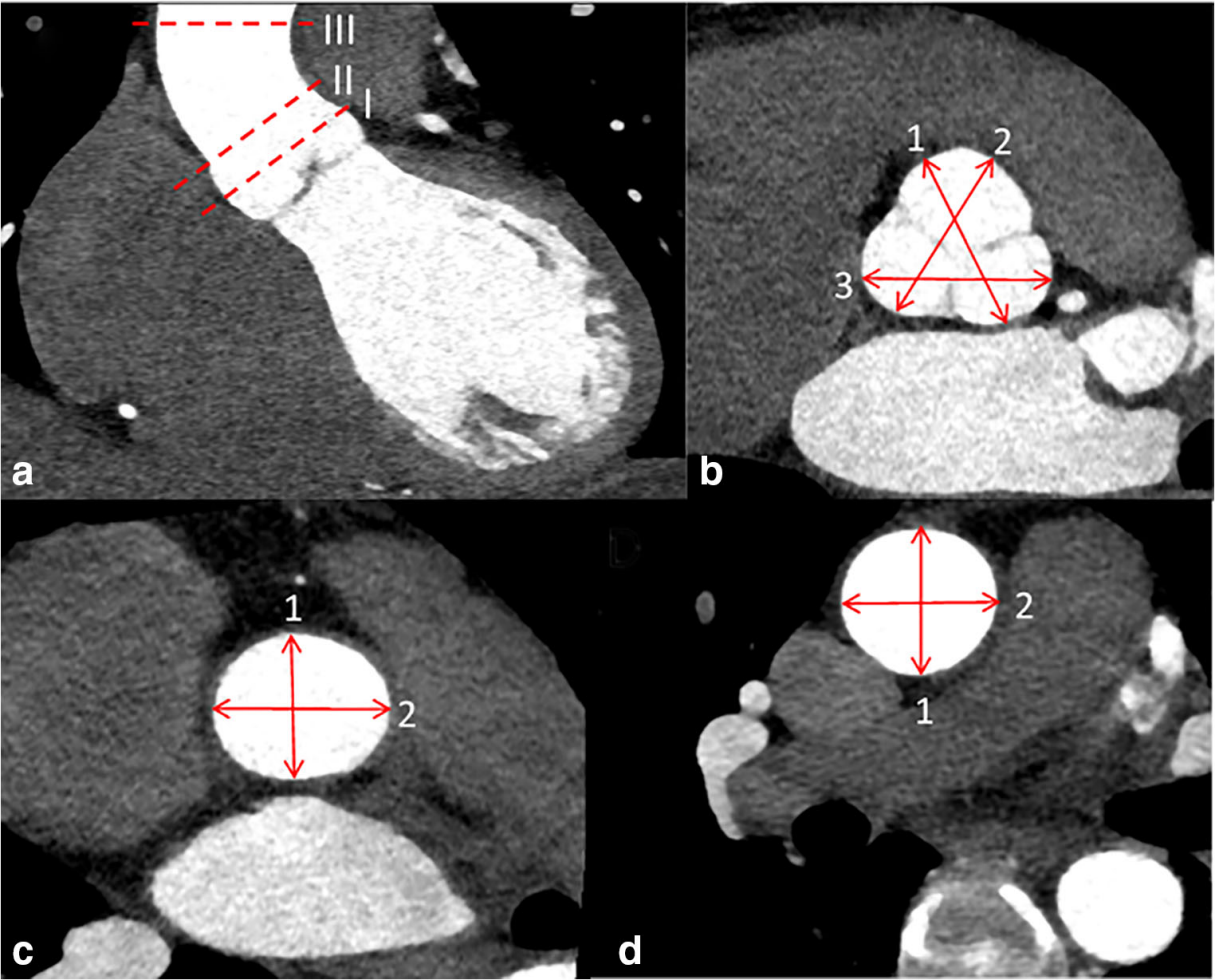
**a** Diameters of ascending aorta were measured in three planes: sinus valsalva (I), sinotubular junction (II), and tubular part (III). **b** The diameter of sinus valsalva was assessed as the largest of the three dome of the cusp to dome of the cusp diameters from the outer layer to the outer layer of the aortic wall (arrows). **c**, **d** The largest of the two diameters (1 and 2) of sinotubular junction and tubular part was measured as perpendicular to each other

One observer retrospectively analyzed the CCTA images on an IDS7 diagnostic workstation (version17.3.6; Sectra Imtec). The slice thickness of the CCTA images was 0.6 mm. The ascending aorta was divided into 3 planes: sinus valsalva, sinotubular junction, and tubular part (Fig. 1). According to current recommendations, aortic diameters were measured from the outer-to-outer vascular wall perpendicular to the centerline of the vessel [1]. The largest of the three dome of the cusp to dome of the cusp diameters in sinus valsalva and the largest of the two diameters in sinotubular junction and tubular part were registered. Aortic valve anatomy (tricuspid, bicuspid, or mechanical aortic valve prosthesis), middle diastolic diameter of cardiac left ventricle (LV), area of left atrium (LA), thickness of left ventricular posterior wall, and interventricular septum were registered.

#### AA dilatation classification methods

Several published classification methods were first applied to assess the frequencies of AA dilatation: the *ESC 2014 recommendations* (ESC Diameter) [1], *Roman’s classification* (aortic size index, ASI) [4], and *aortic height index* (AHI). The thresholds for aortic dilatation with each method were based on the values in the literature. After stratifying patients in the overall population into dilated or non-dilated groups, similar stratifications were made in the subgroup of patients with no risk factors.

In ESC Diameter, the AA was considered dilated if its greatest diameter exceeded 40 mm in any of the three measurement planes [1].

To estimate the ASI, we calculated the relationship between AA and BSA using Mosteller’s equation [16]; $\frac{\mathrm{Aortic}\ \mathrm{diameter}\ \left(\mathrm{mm}\right)}{\mathrm{BSA}\ \left({\mathrm{m}}^2\right)}$ [17]. In our study, data on height was available for 775 patients, and on weight for 740 patients at the time of CCTA. Thus, BSA could be calculated in 740 patients. For both genders, the upper limit for ASI was 21 mm/m^2^ at the sinus valsalva plane according to Roman et al [4].

Furthermore, we calculated AHI to represent the relationship between aortic size and patient height: $\frac{\mathrm{Aortic}\ \mathrm{diameter}\ \left(\mathrm{m}\mathrm{m}\right)}{\mathrm{patient}\ \mathrm{height}\ \left(\mathrm{m}\right)}$, which has been reported to evaluate satisfactorily the risk of complications in patients with ascending aortic aneurysms [18].

Finally, based on aortic diameters and the calculated ASI and AHI values, we arbitrarily defined an upper threshold of two standard deviations (2 SD, 95%) above the mean value as the upper limit of normal aortic diameter in the subgroup of patients with no risk factors. These thresholds were named as Aortic Diameter_2SD_, ASI_2SD_, and AHI_2SD_, and they were subsequently used to calculate the prevalence of increased aortic diameter in the overall study population.

#### Risk factors

Risk factors for cardiovascular diseases as well as other baseline characteristics were collected from medical records. The patient was defined as hypertensive if he/she was receiving medication for hypertension and diabetic if the patient had two separate fasting plasma glucose levels ≥ 7.0 mmol/l or ≥ 11 mmol/l in a glucose tolerance test or HbA_1C_ ≥ 48 mmol/l. Diabetes was not subdivided into subtypes. Current smokers and those who had stopped continuous smoking less than 30 years ago were considered smokers. Based on the coronary artery findings in the CCTA, the patients were dichotomized as positive or negative in terms of CAD. CCTA reports were prepared by imaging cardiologists or cardiac radiologists with over 6 years of experience in cardiac imaging. Hypercholesterolemia was determined according to Finnish national recommendations as high LDL (> 3 mmol/l) and low HDL (males < 1 mmol/l, females < 1.2 mmol /l) concentrations.

### Statistical analysis

The normality of the aortic dimension data was analyzed using the Kolmogorov–Smirnov test. Correlations between the diameters of AA and continuous scaled parameters were tested using Spearman correlation test. Continuous parameters were tested with the Mann–Whitney test with the results being presented as median values with variable range. Chi-square test was used for nominal parameters and results are presented as numbers and percentages. McNemar’s test was used to compare two dependent dichotomic variables. Statistical significance was set to *p* < 0.05 and high statistical significance to *p* < 0.001. All statistical analyses were performed by using SPSS Statistics 23 (IBM).

### Ethical aspects

The study was approved by the local ethical committee. CCTA imaging had been performed on the basis of clinical indications; thus, the study caused no additional radiation dose to the patients. The patients’ clinical treatment was completely unaffected by the study.

## Results

Baseline characteristics are shown in Table 1. The mean age of the overall study population was 52.9 ± 9.8 years and a majority of the patients were women (*n* = 665, 66.5%). The mean age of the subgroup of patients with no risk factors (*n* = 260 females, 71.2%) was 50.1 ± 10.8 years and their mean BSA value was 1.9 ± 0.2 m^2^.

According to the ESC guidelines, 230 patients were stratified as having a dilated AA when the measurement results from all three levels were combined. Thus, the overall prevalence of AA dilatation was 23.0% in the overall study population (8.1% in females and 52.5% in males) (Table 2). The prevalence of dilatation in the area of aortic root ranged from 5.1% in females up to 50.4% in males and in the tubular part from 4.7% in females up to 14.9% in males.

**Table 2:** Prevalence of ascending aortic dilatation (%) determined with different classification methods; ESC (upper limit was set as 40 mm) and upper 2 SD values derived from the subgroup of patients with no risk factors. The overall study population in the upper part of the table and the subgroup with no risk factors in the lower part of the table

Classification method	Gender	Sinus valsalva (%)	Sinotubular junction (%)	Tubular part (%)	Any plane (%)
Overall study population					
ESC [1]	All	20.3	2.3	8.1	23.0
	Males	50.4	4.8	14.9	52.5
	Females	5.1	1.1	4.7	8.1
Diameter_2SD_	All	7.7	8.1	8.5	14.5
	Males	6.6	7.5	8.4	14.9
	Females	6.5	6.0	7.4	12.5
ASI_2SD_	All	5.5	6.6	6.2	10.3
AHI_2SD_	All	8.9	7.7	9.6	15.7
Subgroup of patients with no risk factors					
ESC [1]	All	12.9	0.8	4.4	15.1
	Males	37.1	2.9	7.6	39.0
	Females	3.1	0	3.1	5.4

*Abbreviations*: *AHI*, aortic height index; *ASI*, aortic size index; *ESC*, European Society of Cardiology

According to Roman’s classification, in the 740 patients for whom we had access to their height and weight data in medical records, 163 patients (23.0% of males and 21.5% of females) were stratified as having a dilated aortic root in the sinus valsalva plane. Thus, the prevalence of dilatation was 22.0% in both genders.

When the calculated prevalence of dilatation in the sinus valsalva plane was compared only in the male patients in our consecutive population, the prevalence was significantly higher when the ESC criteria were utilized in comparison to Roman’s method (*p* < 0.001). In contrast, in the female patients, the prevalence was significantly lower when the ESC criteria were compared with Roman’s method (*p* < 0.001).

In the subgroup of patients with no risk factors, the overall prevalence of AA dilatation was 15.1% (5.4% in females and 39.0% in males) according to the ESC recommendations. The prevalence of dilatation in aortic root ranged from 3.1% in females up to 37.1% in males and in the tubular part from 3.1% in females up to 7.6% in males.

Based on the values assessed in the subgroup of patients with no risk factors, thresholds for Aortic Diameter_2SD_, ASI_2SD_, and AHI_2SD_ are presented in Table 3. With all measured planes combined, the frequencies of AA dilatation in the overall study population were 14.5% according to the Aortic Diameter_2SD_, 10.3% when based on ASI_2SD_, and 15.7% when determined with AHI_2SD_ (Table 2).

**Table 3:** Threshold values of the diameters, aortic size index, and aortic height index indicating the upper two standard deviations (2 SD, 95%) of the normally distributed data in the subgroup of patients with no hypertension, coronary artery disease, or bicuspid or mechanical aortic valve

Classification method	Gender	Sinus valsalva		Sinotubular junction		Tubular part		Any plane	
		Mean	2 SD	Mean	2 SD	Mean	2SD	Mean	2 SD
Diameter_2SD_ (mm)	Total	35.9	43.7	29.4	35.9	32.2	39.9	32.5	39.8
	Males	39.0	47.0	31.5	38.4	33.3	42.5	34.6	42.6
	Females	34.6	39.7	28.5	33.8	31.9	38.7	31.7	37.3
ASI_2SD_ (mm/m^2^)	All	19.2	23.2	15.7	19.4	17.3	22.2	17.4	21.6
AHI_2SD_ (mm/m)	All	21.3	24.8	17.4	20.9	19.1	23.3	19.3	23.0

*Abbreviations*: *AHI*, aortic height index; *ASI*, aortic size index

**Fig. 2:**
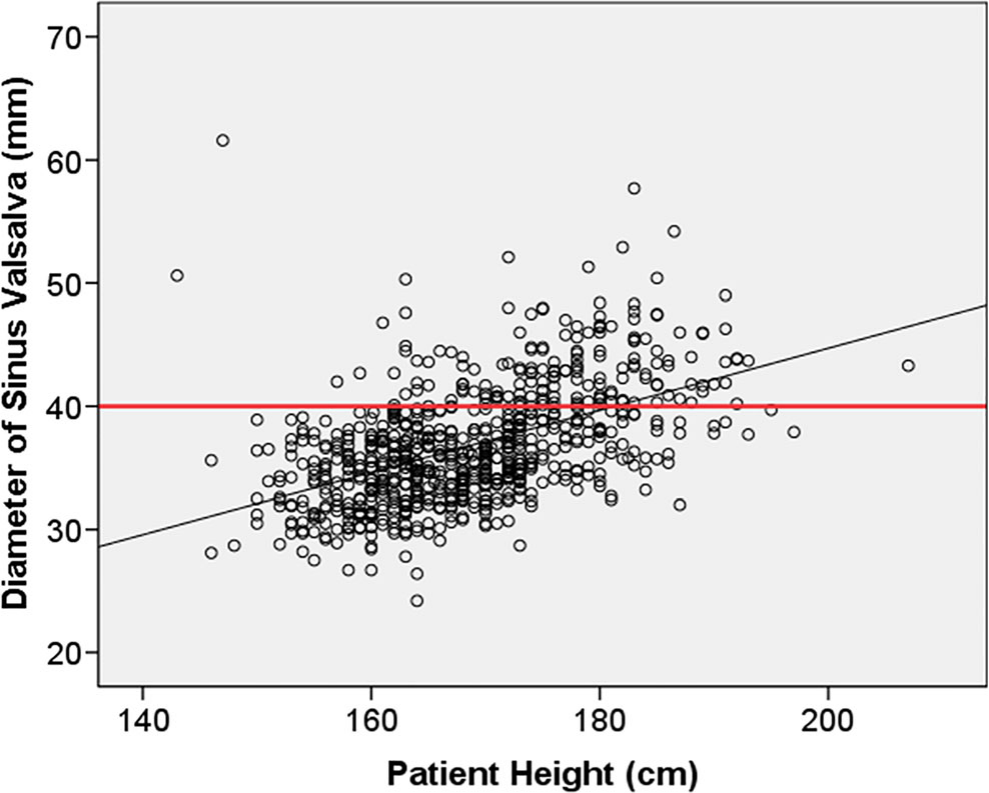
Association between the diameter of the sinus valsalva and the patient’s height. The ESC recommendation for the upper limit of a normal aorta (40 mm) is shown by the red reference line. The linear correlation (*r* = 0.535, *p* < 0.001) between the diameter of the sinus valsalva and the patient’s height is shown by the black line

Associations between the baseline characteristics and clinical risk factors with respect to the prevalence of aortic dilatation are shown in Table 4. According to the ESC criteria, BSA was associated with larger AA diameters in the overall population (*r* = 0.407, *p* < 0.001) and in the subgroup of patients with no risk factors (*r* = 0.405, *p* < 0.001). Furthermore, according to the ESC criteria, male gender was associated with AA dilatation (*p* < 0.001), i.e., 176 males (76.5%) but only 54 females (23.5%) had dilated AA. Other factors associated with dilated AA were hypertension in females (*p* = 0.009), BAV (*p* < 0.001), increased LV wall thickness (*p* < 0.001), increased middle diastolic LV diameter (*p* < 0.001), and increased LA area (*p* < 0.001), Table 4. The association between the diameter of sinus valsalva and the patient’s height is illustrated in Fig. 2. According to the ESC criteria, if the patient was taller than 180 cm, this increased significantly the prevalence of dilatation in his/her sinus valsalva plane (61.8%) compared with patients whose height was less than 180 cm (16.8%, *p* < 0.001).

**Table 4:** Prevalence of cardiovascular risk factors and their association with the presence of aortic dilatation in the overall study population. Prevalence and association were calculated separately based on the ESC 2014 guidelines for aortic diseases and by using ASI_2SD_ and AHI_2SD_ where the thresholds were assessed from our subgroup of patients with no hypertension, coronary artery disease, and bicuspid or mechanical aortic valve

	ESC recommendation (*n* = 1000)		ASI_2SD_ (*n* = 740)				AHI_2SD_ (*n* = 732)		
	Dilated (*n* = 230)	Non-dilated (*n* = 770)	*p* value	Dilated (*n* = 76)	Non-dilated (*n* = 664)	*p* value	Dilated (*n* = 115)	Non-dilated (*n* = 617)	*p* value
Male gender (%)	176 (76.5)	159 (20.6)	< 0.001	20 (26.3)	236 (35.5)	0.109	58 (50.4)	194 (31.4)	< 0.001
Female gender (%)	54 (23.5)	611 (79.4)	< 0.001	56 (73.7)	428 (64.5)	0.109	57 (49.6)	423 (68.6)	< 0.001
Hypertension (%)	116 (50.4)	339 (44.0)	0.087	38 (50.0)	328 (49.4)	0.921	63 (54.8)	300 (48.6)	0.225
Smoking (%)	85 (37.0)	169 (21.9)	< 0.001	15 (19.7)	194 (29.2)	0.082	36 (31.3)	171 (27.7)	0.433
Over 50% stenosis in CCTA (%)	38 (16.5)	111 (14.4)	0.070	15 (19.7)	108 (16.3)	0.317	17 (14.8)	104 (16.9)	0.755
Coronary calcification in CCTA (%)	74 (32.2)	152 (19.7)	< 0.001	21 (27.6)	159 (23.9)	0.342	42 (36.5)	137 (22.2)	0.001
Bicuspid aortic valve (%)	23 (10.0)	8 (1.0)	< 0.001	13 (17.1)	15 (2.3)	< 0.001	19 (16.5)	8 (1.3)	< 0.001
Height (cm)	177.0 (171.0–183.0)	165.0 (160.0–172.0)	0.005	163.0 (158.3–171.8)	169.0 (162.0–175.0)	< 0.001	171.0 (162.0–178.0)	168.0 (161.5–174.0)	0.128
Weight (kg)	88.0 (78.4–100.0)	75.0 (65.0–85.8)	< 0.001	64.5 (57.0–74.8)	80.0 (69.0–92.0)	< 0.001	82.3 (71.8–91.0)	77.0 (66.0–89.2)	0.013
BSA (m^2^)	2.1 (2.0–2.2)	1.9 (1.7–2.1)	< 0.001	1.7 (1.6–1.9)	1.9 (1.8–2.1)	< 0.001	2.0 (1.8–2.1)	1.9 (1.7–2.1)	0.013
Left ventricular diastolic diameter (mm)	51.0 (46.0–56.0)	46.0 (44.0–50.0)	< 0.001	47.0 (43.5–52.0)	48.0 (44.0–52.0)	0.473	50.0 (46.0–56.0)	47.0 (44.0–51.0)	0.001
Area of left atrium (mm^2^)	19.0 (17.0–24.0)	16.0 (14.0–19.0)	< 0.001	16.0 (14.8–21.0)	17.0 (15.0–20.0)	0.564	19.0 (16.0–24.0)	17.0 (15.0–19.0)	< 0.001
Left ventricular posterior wall thickness (mm)	9.0 (8.0–11.0)	8.0 (8.0–9.0)	< 0.001	9.0 (8.0–10.0)	9.0 (8.0–10.0)	0.997	9.0 (8.0–10.0)	9.0 (8.0–10.0)	0.001
Interventricular septum thickness (mm)	11.0 (9.0–12.0)	9.0 (8.0–10.0)	< 0.001	10.0 (9.0–10.0)	10.0 (9.0–11.0)	0.914	10.0 (9.0–11.8)	10.0 (9.0–11.0)	0.002

Results are presented as numbers and percentages for nominal parameters (chi-square test) and median and range for continuous parameters (Mann–Whitney test)

*Abbreviations*: *BSA*, body surface area; *CCTA*, coronary computed tomography angiography

When using the ASI_2SD_ classification, gender was not associated with the prevalence of AA, but using AHI_2SD_, gender was associated with AA dilatation (*p* < 0.001) (Table 4). Also, increased LV wall thicknesses (*p* = 0.001), LV middle diastolic diameter (*p* = 0.001), and increased LA area (*p* < 0.001) were associated with AA dilatation using AHI_2SD_ classification (Table 4).

## Discussion

When applying the current ESC guidelines for aortic dilatation, the prevalence of AA dilatation was as high as 23% in our consecutive CCTA population. In males, this prevalence exceeded 50% and in male patients taller than 180 cm, the prevalence exceeded 60%. Even in those patients without hypertension, coronary artery disease, or bicuspid or mechanical aortic valve, the prevalence still remained as high as 15%. Our results indicate that the ESC guidelines may overestimate the prevalence of aortic dilatation and might lead to unwarranted distress in patients who may be subjected to unnecessary follow-up examinations by either CT or MRI. Furthermore, a greater BSA is moderately associated with larger AA diameters. BSA-adjusted classification is a level of evidence B in ESC guidelines. Thus, when diagnosing aortic dilatation, it might be better to use BSA-adjusted classifications.

Aortic dilatation is one of the main causes for repeated imaging and clinical follow-up. The additional follow-up imaging caused by unreliable classification levels burdens the healthcare system and increases costs. The mean age of our patient cohort was 53 years indicating that every second male patient would require follow-up imaging for decades should the ESC recommendations be followed. In general, the limits for AA dilatation should be based on the risk for aortic complications: rupture, dissection, or death. The incidence of rupture of thoracic aortic aneurysm is 5/100,000 (0.005%) [19]. Davies et al demonstrated that thoracic aortic diameters in the range 35–39 mm are not associated with aortic ruptures or dissections [20]. However, in that same study, a thoracic aortic diameter of 60 mm or over was related to a dramatic risk of either rupture or dissection, i.e., the odds ratio for rupture increased by 27-fold.

The incidence of AA dilatation in the North American population has been shown to be approximately 10/100,000 inhabitants (0.01%) and it was similar in both genders [21]. Compared with these previous data, the prevalence values estimated in our study population seem extremely high.

ESC guidelines do not present rigorous thresholds to the upper limit of AA diameter, but it states that aortic diameters do not normally exceed 40 mm in healthy adults, which is based on published data [1].

Previously, Roman et al have suggested that the upper limit for normal sinus valsalva diameter would be 21 mm/m^2^ using ASI [4]. They calculated this limit on the basis of a study population consisting of 135 adults. The corresponding upper limit in our study was slightly higher (23 mm/m^2^) when using the ASI_2SD_ threshold determined from our subgroup of patients with no risk factors. For comparison, in their MRI study, Mensel et al postulated that the normal upper limit for the tubular part would be 42 mm for males and 39 mm for females [22]. Our results parallel the thresholds published by Mensel et al as the normal upper limit for tubular part in our study was 43 mm for males and 39 mm for females when using the AA diameter_2SD_ assessment. Mensel et al did not examine the aortic root diameters [22].

Similar to previous reports, the associated risk factors for AA dilatation in the present study were male gender, BAV, hypertension, and smoking [4–6]. Hypertension is only associated with AA dilatation in males. The diameters of sinus valsalva and tubular part also correlated with the patient’s height, weight, and therefore also with BSA. BSA has also been observed to correlate positively with AA dilatation [4, 6, 23]. In the present study, increased LV wall thickness was also associated with aortic dilatation when applying the ESC recommendations. Aortic root dilatation may lead to aortic regurgitation and it has also been reported to be associated with LV hypertrophy, LV dilatation, and LV dysfunction [24]. However, when using ASI_2SD_ thresholds, LV thickness is not associated with AA dilatation.

Male gender is associated with high statistical significance to AA dilatation when using 40 mm as the upper limit of a normal AA in agreement with previous studies [6]. However, gender is not associated with the prevalence of AA dilatation when using the ASI_2SD_ method due to the fact that BSA is strongly linked with gender (i.e., men tend to be taller and heavier than women).

The main limitations of this retrospective study were the higher number of females and that only a limited part of the AA was available for analysis due to imaging stack disposition. In addition, a relatively high number of patients had risk factors for cardiovascular diseases. However, all patients had only a low to moderate pretest probability for CAD and the patients’ mean age was 52.9 years with a relatively low standard deviation (9.8 years), which means that this population was representative of clinical CCTA populations in many hospitals. Intra- and interobserver reproducibility analyses for measurements of AA diameter were not performed in the present study. However, in our prior study with 1.5-T aortic magnetic resonance angiography, interobserver reproducibility was shown to be excellent (ICC = 0.917) with 20 dilated AA and 20 non-dilated AA patients [7]. Lu et al showed in a small 30-patient CT population that the maximum variability was 1.2 mm in the measured AA diameter and low variability indicated that AA diameter is a reliable measurement [25]. ECG-gated CT is an important tool for precise measurements of AA diameter.

In conclusion, the prevalence of thoracic aortic dilatation proved to be relatively high in this consecutive CCTA population if they were assessed with the ESC 2014 guidelines. This has significant clinical consequences, since patients whose values lie outside the normal limits are usually scheduled for repeated follow-up for the rest of their lives. Based on this study and ESC guidelines, we propose that a more reliable way to evaluate thoracic aortic diameter would be indexing the AA diameter to body size by calculating it in terms of BSA. By using indexed upper limits for AA, the specificity of the stratifications would increase, and repeated follow-up might be targeted to those patients who will truly benefit. However, more clinical studies will be needed to determine the optimal normal limits for AA dilatation in different patient populations.

## Abbreviations

2 SD: Two standard deviations
AA: Ascending aorta
AHI: Aortic height index
ASI: Aortic size index
BAV: Bicuspid aortic valve
BSA: Body surface area
CAD: Coronary artery disease
CCTA: Coronary computed tomography angiography
CT: Computed tomography
ESC: European Society of Cardiology
LA: Left atrium
LV: Left ventricle
MRI: Magnetic resonance imaging
TAD: Thoracic aortic dilatation
